# Multiple *MYO18A*-*PDGFRB* fusion transcripts in a myeloproliferative neoplasm patient with t(5;17)(q32;q11)

**DOI:** 10.1186/s13039-017-0306-8

**Published:** 2017-02-27

**Authors:** Guangying Sheng, Zhao Zeng, Jinlan Pan, Linbing Kou, Qinrong Wang, Hong Yao, Lijun Wen, Liang Ma, Depei Wu, Huiying Qiu, Suning Chen

**Affiliations:** 1 Jiangsu Institute of Hematology, Key Laboratory of Thrombosis and Hemostasis of Ministry of Health, the First Affiliated Hospital of Soochow University, 188 Shizi Street, Suzhou, Jiangsu province 215006 China; 2 Collaborative Innovation Center of Hematology, Soochow University, Suzhou, China

**Keywords:** PDGFRB rearrangement, MYO18A, MPNs, Imatinib

## Abstract

**Background:**

Myeloproliferative neoplasms (MPNs), typically defined by myeloid proliferation and eosinophilia, and are only rarely caused by platelet-derived growth factor receptor beta (PDGFRB) gene rearrangements.

**Case presentation:**

Here, we report a unique case of MPN that is negative for eosinophilia and characterized by a novel *PDGFRB* rearrangement. After cytogenetic analysis revealed a karyotype of t(5;17) (q32;q11), we used fluorescence *in situ* hybridization to specifically identify the *PDGFRB* gene at 5q31-q33 as the gene that had been translocated. Subsequently, RNA sequencing identified a new *MYO18A*-*PDGFRB* gene fusion. This fusion presented a previously undescribed breakpoint composed of exon 37 of *MYO18A* and exon 13 of *PDGFRB*. Furthermore, both RT-PCR and Bi-directional Sanger sequencing confirmed this out-of-frame fusion. Interestingly, we simultaneously identified the presence of another three *PDGFRB* transcripts, all of which were in-frame fusions. After treating the patient with imatinib, the t(5;17) translocation was no longer detected by conventional cytogenetics or by FISH, and at the time of the last follow-up, the patient had been in complete remission for 26 months.

**Conclusion:**

We prove that *MYO18A*-*PDGFRB* fusions are recurrent genetic aberrations involved in MPNs, and identify multiple fusion transcripts with novel breakpoints.

## Background

Constitutive activation of protein tyrosine kinases is a common feature of the pathogenesis of chronic myeloproliferative neoplasms (MPNs). The genes most commonly involved in these neoplasms are those encoding for the protein tyrosine kinases PDGFRA, PDGFRB, FGFR1, KIT, FLT3, JAK2 and ABL1. In 2008 however, the World Health Organization (WHO) classified rearrangements of *PDGFRA*, *PDGFRB*, and *FGFR1* in a distinct disease category [1]. PDGFRB is a class III receptor tyrosine kinase located on chromosome 5 at position 5q31-q33. The most common type of *PDGFRB* aberration is a fusion translocation. To date, more than 30 different *PDGFRB* fusion partners have been identified [2, 3], with the majority of them only occurring in individual patients. Nonetheless, a few of these fusions - for example, *ETV6-PDGFRB*, *H4-PDGFRB*, and *CCDC88C-PDGFRB* [2, 4–6] – are in fact recurrent. Interestingly, each partner typically contains an oligomerization motif that contributes to protein dimerization and consequently to the constitutive activation of the PDGFRB kinase domain. Imatinib, a tyrosine kinase inhibitor typically used to treat those myeloid tumors characterized by *PDGFRB* fusions, is reported to produce sustained remission in nearly all cases [2, 3].

MYO18A, a member of the myosin superfamily originally identified in bone marrow stromal cells, is associated with the ability of these cells to support hematopoiesis [7]. In hematological malignancies, *MYO18A* has been found as fusions with *FGFR1*, *PDGFRB*, and in only a single case, with *MLL*, leading to the 8p11 myeloproliferative syndrome (EMS), eosinophilia-associated MPN (MPN-eo), and acute myeloid leukemia (AML) respectively [8–10].

Here, we present a novel case of MPN in which a unique *MYO18A*-*PDGFRB* fusion results in MPN without eosinophilia. To the best of our knowledge, this is the first time that fusion to exon 13 of *PDGFB* has been reported. Interestingly, this patient harbored multiple *MYO18A-PDGFRB* transcripts, with most of them being in-frame fusions. Therapeutically, this patient was sensitive to imatinib, and achieved both complete hematological remission (CHR) and complete cytogenetic remission (CCyR) in a sustained and rapid manner.

## Case presentation

A 25-year-old man was admitted to our medical center after experiencing prolonged weakness and splenomegaly. A complete blood cell analysis indicated a white blood cell count of 81,190/μL, a platelet count of 206,000/μL, and a hemoglobin concentration of 10.5 g/dL. Peripheral blood analysis showed all stages of neutrophilic maturation, with 1% myeloblasts, 4% promyelocytes, 10% myelocytes, 17% metamyelocytes, 2% eosinophils and 9% basophils. Bone marrow aspirates revealed that the patient was in the chronic phase of chronic myeloid leukemia (CML), with 2.5% myeloblasts, 5.5% promyelocytes, 13% myelocytes, 13% metamyelocytes, 1.5% eosinophils and 9% basophils. A multiplex PCR screen for gene fusions typical of leukemia was negative. Chromosome analysis of the bone marrow showed the presence of 46,XY,t (5;17) (q32;q11) [10]. Subsequently, dual-color fluorescent *in situ* hybridization (FISH) proved positive for *PDGFRB* gene rearrangement. After one week of imatinib treatment at 400 mg/day, the patient achieved CHR. This dosage was then decreased to 200 mg/day, and the patient acquired CCyR three months later. Thereafter, the patient took 100 mg/day, and at the final follow-up (26 months later), the patient still remained in complete remission.

## Results

**Fig. 1:**
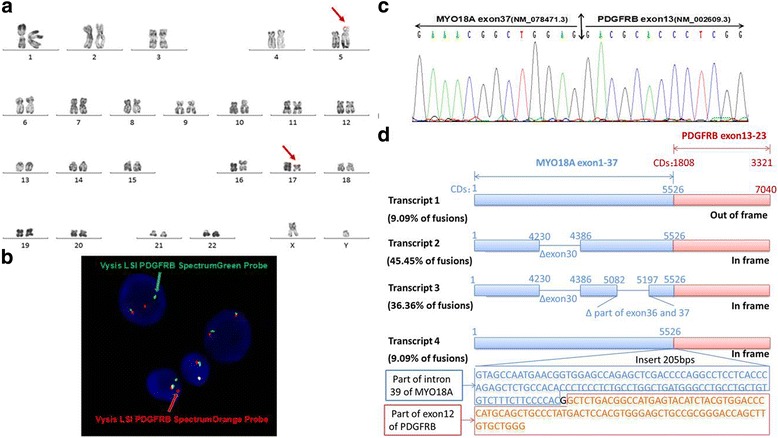
Multiple *MYO18A*-*PDGFRB* fusion transcripts in a myeloproliferative neoplasm patient with t(5;17) (q32;q11) (**a**) The R-banded karyotype showing the translocation t(5;17) (q32;q11). The arrows indicate the structural aberrations of chromosomes 5 and 17; (**b**) FISH using Vysis LSI PDGFRB Spectrum Green and Orange Probes. A yellow signal denotes a normal *PDGFRB* gene (5’and 3’regions remain joined), whereas orange and green signals respectively denote the 5’ and 3’ regions of the *PDGFRB* gene after rearrangement; (**c**) Bi-directional Sanger sequencing of the PCR product confirms the fusion between *MYO18A* exon 37(NM_078471.3) and *PDGFRB* exon 13(NM_002609.3); (**d**) Four distinct types of *MYO18A*-*PDGFRB* fusion transcripts were detected in the patient

Through retrospective analysis, we identified an MPN patient with a karyotype of 46,XY,t(5;17) (q32;q11) (Fig. 1a). This suggested a rearrangement of the *PDGFRB* gene located at chromosome 5q31-q33. In order to confirm the suspected rearrangement, we performed FISH analysis using two distinct probes complementary to the 5’ and 3’ regions of the *PDGFRB* gene, and found separated signals in 75% of blast cells (Fig. 1b). A previous study by Walz et al. found that translocation events at 17q11 were associated with the *MYO18A* gene [9]. RT-PCR using *MYO18A* (exon 40) and *PDGFRB* (exon 10) primers was unable to detect this previously reported *MYO18A*-*PDGFRB* fusion transcript (data not show) [9]. Whole transcriptome sequencing, however, uncovered a fusion between exon 37 of *MYO18A* (NM_078471.3) and exon 13 of *PDGFRB* (NM_002609.3). Subsequently, RT-PCR and Bi-directional Sanger sequencing confirmed this out-of-frame fusion between *MYO18A* exon 37 and *PDGFRB* exon 13 (Fig. 1c). Given that the patient was highly sensitive to imatinib-mediated PDGFRB inhibition, but showed no other imatinib-related abnormalities, we hypothesized that there was some degree of splicing taking place within the *MYO18A*-*PDGFRB* fusion. In fact, we were able to amplify different products containing the same fusion point by RT-PCR, and a total of four types of *MYO18A*-*PDGFRB* fusion transcripts were identified by Sanger sequencing (Fig. 1d). Three out of four of these transcripts were actually in-frame *PDGFRB* fusions and accounted for a total of 90.9% of the identified transcripts (Fig. 1d). Two of the in-frame transcripts contained a deletion of *MYO18A* exon 30, with one of them also having parts of exons 36–37 deleted at the same time. Finally, the third in−frame fusion transcript had an insertion including part of *MYO18A* intron 39 and part of *PDGFRB* exon 12 (Fig. 1d).

## Discussion and Conclusion

Although *PDGFRB* fusions are rare, they can be observed in a diverse range of hematological malignancies including Ph-like acute lymphoblastic leukemia (ALL), AML, and atypical CML. These fusions have an adult male predominance, and are clinically defined by eosinophilia and splenomegaly [2]. To date, more than 30 partner genes of *PDGFRB* have been identified [2, 3]. However, only a minority of these are recurrent (the most common of which is *ETV6*-*PDGFRB*), with the vast majority being reported in only individual cases. Walz et al. reported the first eosinophilia-associated MPN attributed to a *MYO18A*-*PDGFRB* fusion in 2009 [9]. Here, we report another case of MPN that is characterized by unique *MYO18A*-*PDGFRB* fusions and an eosinophilia-free phenotype. In agreement with other studies, this suggests that eosinophilia is a prominent, but not invariable feature [11].

Interestingly, in the present case exon 37 of *MYO18A* became fused in an out-of-frame fashion to exon 13 of *PDGFRB*. However, as the patient was highly sensitive to imatinib treatment - a pharmaceutical drug which inhibits activated receptor tyrosine kinases including PDGFRB - and displayed no other abnormalities, we suspected that the patient harbored different spliced versions of the fusion. Indeed, RT-PCR and sequencing enabled us to identify four types of fusion transcripts in our patient, with three of them being in-frame fusions. Interestingly, *PDGFRB* fusions principally involve exons 11 and 12, and more rarely exons 9 and 10 [9, 12]. In fact, there is no previous report of exon 13 being involved in the fusion point, possibly due to it being an out-of-frame fusion. The in-frame MYO18A-PDGFRB fusion proteins included the majority of the domains of MYO18A, including some of the coiled-coil motifs, and the entire tyrosine kinase domain of PDGFRB. It is known that only one, or a few of these coiled-coil motifs are enough to promote the dimerization or oligomerization of MYO18A-PDGFRB fusions. As in the case of the MYO18A-FGFR1 fusion, this leads to constitutive activation of the tyrosine kinase domain [8]. In addition, the lack of the PDGFRB transmembrane domain, which is found in exon 11, results in delocalization of the fusion protein to the cytoplasm. This may be another mechanism through which the kinase domain becomes constitutively activated. Furthermore, deletion of exon 12, which results in the disruption of the autoinhibitory WW-like domain of the juxtamembrane region, may also promote transformation properties of such fusion proteins [13–15]. Of course, however, loss of these domains is not mandatory for transformation because in previous cases, other MYO18A-PDGFRB fusions retained both the transmembrane and juxtamembrane domains.

Although *PDGFRB* fusions are rare, the identification of *PDGFRB* rearrangements is significant because of their sensitive response to imatinib, especially with respect to MPNs [16]. Moreover, both primary and secondary resistance to imatinib seems to be very rare [17, 18]. Due to its low incidence, a standard imatinib dose and medication time for patients with *PDGFRB* fusions has yet to be formally established in clinical treatment. With the standard dosage used for CML as a reference, patients are initially treated with 400 mg/day [16, 19]. In fact, PDGFR fusions are much more sensitive to imatinib than BCR-ABL1 fusions, at least *in vitro*. For instance, the IC50 of imatinib for inhibiting the proliferation of ETV6-PDGFRB transformed cells is 7.5 nM, a value significantly lower compared to the IC50 of 800 nM for BCR-ABL transformed cells [20, 21]. In our case, imatinib at 400 mg/day brought about CHR in only one week, and after decreasing the dosage, CCyR was also obtained within three months. Thereafter, complete remission remained stable, indicating that a low dose of imatinib is appropriate for patients with *PDGFRB* fusions.

Overall, our findings further emphasize the prominent role of *PDGFRB* in the pathogenesis of MPNs, and highlight the importance of accurate diagnosis and targeted therapy.

## Methods

### R-banding and karyotyping

At the time of diagnosis, the bone marrow cells were cultured for 24 h and analyzed for standard cytogenetic R-banding. The karyotype was described according to the International System for Human Cytogenetic Nomenclature (ISCN 2013).

### Fluorescence *in situ* hybridization (FISH) analysis

FISH analysis was performed on metaphase spreads, using the Vysis LSI PDGFRB Spectrum Orange and Green Probes (Abbott Molecular, Illinois, USA) according to the manufacturer’s instructions.

### RNA sequencing

Whole transcriptome sequencing of bone marrow mononuclear cells was performed, and transcription sequence data were generated by high-throughput RNA sequencing (Illumina HiSeq 2500). The online software deFuse was used for the discovery of fusion transcripts.

### RT-PCR and Sanger sequencing

RNA was reverse transcribed with random hexamers using standard techniques (Thermo Fisher), and the following primer sets were used to detect *MYO18A*-*PDGFRB* transcripts: MYO18A(c.5165) Forward (5′-ACATCGCCAAAGCCAAGA-3′) and PDGFRB(c.2014) Reverse (5′-GATGGGTCCTCCTTTGGT-3′) at an annealing temperature of 58 °C over 32 cycles; MYO18A(c.2524) Forward (5′-CTGGCGTTTGACGACTTG-3′) and PDGFRB(c.2739) Reverse (5′-CCGTTTGATGGCATTGTAG-3′); and MYO18A(c.5436) Forward (5′-GGACAAGTCCCTGGTGAGC-3′) and PDGFRB(c.3301) Reverse (5′-CTACAGGAAGCTATCCTCTGC-3′) at an annealing temperature of 55 °C over 35 cycles. PCR products were analyzed by Bi-directional Sanger sequencing.

## Abbreviations

ALL: acute lymphoblastic leukemia
AML: acute myeloid leukemia
CCyR: complete cytogenetic remission
CHR: complete hematological remission
CML: chronic myeloid leukemia
EMS: the 8p11 myeloproliferative syndrome
FISH: fluorescence *in situ* hybridization
MPN-eo: eosinophilia-associated MPN
MPNs: myeloproliferative neoplasms
PDGFRB: platelet-derived growth factor receptor beta
WHO: World Health Organization
